# In bromeliad phytotelma, anthropic disturbance does not affect the nematode trophic structure

**DOI:** 10.21307/jofnem-2020-101

**Published:** 2020-11-10

**Authors:** Alexandre Macedo Almeida, Janeo Eustáquio Almeida Filho, Ricardo Moreira Souza

**Affiliations:** 1 Grupo de Pesquisa em Nematologia, Universidade Estadual do Norte Fluminense Darcy Ribeiro, Avenida Alberto Lamego, 2000, Campos dos Goytacazes (RJ), 28015-602, Brazil; 2 Bayer do Brasil. Av. Fernando Osório, 2158, Pelotas (RS), 96065-000, Brazil

**Keywords:** Anthropic disturbance, Anthropogenic disturbance, Atlantic forest, Ecology, Environmental bioindicator, Nematode trophic structure, Phytotelma, Phytotelmata, Tank-forming bromeliad

## Abstract

Phytotelmata (sing. phytotelma) are plant-associated reservoirs of rainwater and organic debris. These freshwater ecosystems are found in tree and bamboo holes, pitcher plants, and tank-forming bromeliads. Some studies suggest that anthropic disturbance (AD) may change the physico-chemical properties (PCPs) of the water retained in the phytotelma, and indirectly impact its biota. Hence, new AD-bioindicators could be found in the phytotelma biota. To test this hypothesis, three areas of Atlantic Forest were selected, distinct only by the level of long-term AD. In these areas, we monitored the nematode trophic structure and the water PCPs in the bromeliad *Neoregelia cruenta* during two years (eight seasons). Significant differences among areas were found in some seasons for total nematode abundance and/or the abundance of some trophic groups, but no pattern emerged relative to the level of AD. Anthropic disturbance did not impact nematode trophic structure possibly because the water PCPs remained fairly similar in all three areas. Our results do not corroborate previous reports that AD alters phytotelma water. On the other hand, our findings support previous studies suggesting that nematodes inhabiting bromeliad phytotelma are not good candidates for AD-bioindicators.

Anthropic disturbance (AD) is a major threat to forest ecosystems, primarily through unmanaged wood harvesting, deforestation, and fragmentation. Microorganisms, micro- and mesofauna have been intensively studied as bioindicators of AD and post-AD restoration. Most studies have focused on benthic and soil communities and ecotoxicology assessments (Mummey et al., 2002; Fränzle, 2006; Verchot, 2010; Balsamo et al., 2012; Shah et al., 2013; Lindenmayer et al., 2015; Sun et al., 2017).

Nematodes have been widely used as bioindicators (Yeates, 2003; Wilson and Kakouli-Duarte, 2009; Hägerbäumer et al., 2015). In terrestrial ecosystems, the response of nematodes to AD has often been sought in the soil, despite indications that, at least in tropical biomes, nematodes are more abundant and diverse aboveground (Porazinska et al., 2010). Two aboveground environments which harbor large, highly diverse, and little-studied nematode communities are the forest canopy and phytotelmata (sing. phytotelma). Phytotelmata are reservoirs of freshwater and organic debris, found in tree and bamboo holes, pitcher plants and tank-forming bromeliads.

Nematodes have been known to inhabit phytotelmata since the 1920s, but only recently have ecologists investigated them in tree holes (Devetter, 2004), plastic cups mimicking tree holes (Ptatscheck and Traunspurger, 2014, 2015; Ptatscheck et al., 2015), and bromeliads (Robaina et al., 2015; Zotz and Traunspurger, 2016; Almeida and Souza, 2020). These studies have shown that in all phytotelmata, bacterial, and hyphal feeder nematodes predominate over plant feeders, unicellular eukaryote feeders and carnivores. Seasonal changes in nematode abundance (total and per trophic group) have been reported in some studies (Ptatscheck and Traunspurger, 2015; Robaina et al., 2015; Zotz and Traunspurger, 2016), but not in others (Ptatscheck and Traunspurger, 2014; Almeida and Souza, 2020).

There is no clear understanding of which environmental factors impact phytotelma nematodes. The amount of organic debris impounded in the phytotelma and the biomass of algae living in the phytotelma water correlate positively with nematode abundance and diversity. Fluctuations in rainfall do not impact the nematodes, while the mean air temperature may affect nematodes in temperate regions, but not in tropical ones (Almeida and Souza, 2020; Ptatscheck and Traunspurger, 2015).

Since phytotelmata are freshwater ecosystems, one should expect the water physico-chemical properties (PCPs) to impact the inhabiting biota. Indeed, these water PCPs have been found to impact communities of algae, archaea, bacteria, micro- and macroinvertebrates (Goffredi et al., 2011; Marino et al., 2011, 2013; Carrias et al., 2012, 2014; Gossner et al., 2016; Louca et al., 2016, 2017). Nonetheless, in nature reserves in Germany and Brazil, the water PCPs had no impact on nematode abundance, except for the positive correlation between the amount of dissolved oxygen and the abundance of hyphal feeder nematodes (Almeida and Souza, 2020; Ptatscheck and Traunspurger, 2014, 2015).

Although natural changes in the water PCPs have no impact on the abundance of phytotelma nematodes, AD may change this pattern. Anthropic disturbance is known to reduce bromeliad species richness and diversity (Rocha et al., 2004, 2007). Wood harvesting, deforestation, and fragmentation expose bromeliads to stronger insolation, higher air temperatures and battering by wind.

Hence, we hypothesized that AD might change the PCPs of the water retained in the bromeliad tanks; and that such changes might impact the nematode trophic structure. Corroboration of this hypothesis would mean that nematodes dwelling in tank-forming bromeliads could be useful bioindicators of AD or post-AD restoration in bromeliad-rich ecosystems.

To test this hypothesis, we investigated bromeliad-rich areas of the Atlantic Forest biome that have been submitted to distinct degrees of AD. In these areas, we monitored the water PCPs and the nematode trophic structure during a two-year period, and sought correlations between those parameters and the level of AD.

## Materials and methods

### Sampling areas

The study was conducted in Restinga de Jurubatiba National Park (RJNP) (http://www.icmbio.gov.br/parnajurubatiba/), in the state of Rio de Janeiro, Brazil. The park protects a large area of *restinga*, an Atlantic Forest ecosystem that includes beaches, dunes, lagoons and areas subject to periodic flooding, in which herbaceous and shrubby species predominate. The region’s climate is AW (tropical savanna) according to the Köppen classification, with an average annual temperature of 23°C and a yearly rainfall ranging from 1,000 to 1,350 mm.

**Figure 1:**
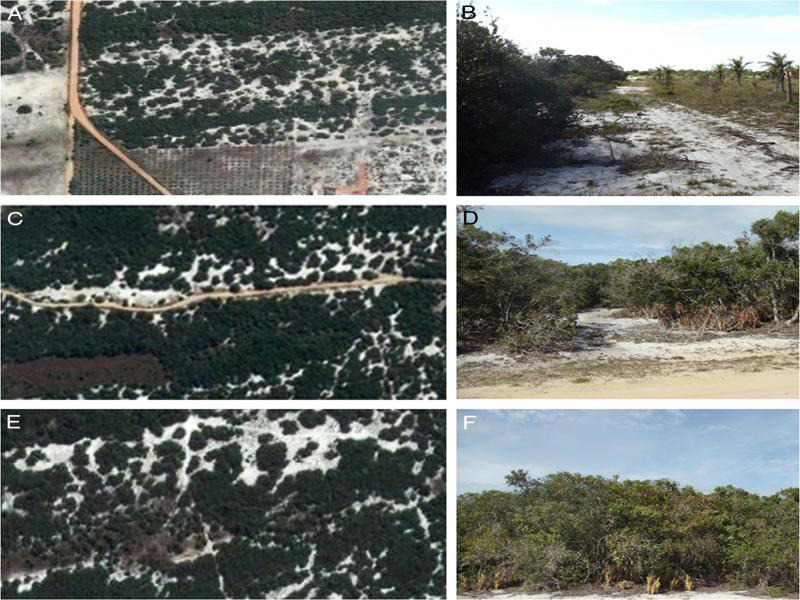
Satellite (source Google Maps^®^) and ground views of areas with different levels of anthropic disturbance, in Restinga de Jurubatiba National Park, Brazil. A, B: area 1, most disturbed; C, D: area 2, intermediate; D, E: area 3, least disturbed.

The RJNP encompasses areas which have been impacted by human activities before the park’s creation. Areas 1, 2, and 3 were selected according to their level of disturbance (Table 1). Area 1 (22°11′09.3″ S; 41°25′50.8″ W) suffered removal of vegetation and impact from a nearby coconut cultivation. Now and then cattle trespass the park’s limits and graze in the area (Fig. 1A, B). Area 2 (22°10′34.7″ S; 41°24′42.0″ W) is located 4 Km away from area 1 and it has suffered relatively less impact. Occasional cattle grazing occurs during the dry season only (Fig. 1C, D). Area 3 (22°09′54.1″ S 41°24′03.1″ W) is located 5 Km away from area 2 and suffered no disturbance, except for the occasional transit of the park’s surveillance vehicles along narrow trails (Fig. 1E, F). These areas are roughly rectangular, ranging from 500 to 600 m × 200 to 300 m (10-18 ha).

**Table 1.** Description and level of anthropic disturbance in three areas of Restinga de Jurubatiba National Park, Brazil^a^.

	Levels		
Description	Area 1	Area 2	Area 3
Removal of vegetation due to road construction	1^b^	1	0
Removal of vegetation for the establishment of commercial activities	1	0	0
Removal of vegetation of gardening/landscaping interest	2	1	0
Removal of vegetation for establishment of coconut plantation	2	0	0
Damage to vegetation due to human trampling	2	2	0
Littering on vegetation	2	1	0
Traffic of vehicles in the area	2	1	1
Establishment of housing	2	0	0
Sand removal for building purposes	2	1	0
Sum	16	7	1

**Notes:** ^a^Adapted from Rocha et al. (2004); ^b^levels: 0: non-existent; 1: little or localized; 2: intense or widespread.

### Sampling and sample processing

Samples were collected in the terrestrial bromeliad *Neoregelia cruenta* (R. Graham) L. B. Smith, which thrives abundantly in the RJNP, shaded by short trees and shrubs. Only mature plants with inflorescence were sampled because this phenological stage is known to support a more abundant nematode community (Almeida and Souza, 2020).

In areas 1, 2, and 3, samplings were carried out from June 2014 through March 2016, once every season, for a total of eight sampling dates. On each sampling date, in each area, eight bromeliads were chosen along a random path. This process was carefully done not to repeat previous paths. The water retained in the bromeliads was suctioned and submitted to the following measurements: volume (mL); temperature (^o^C); pH; dissolved oxygen (DO_2_, in mg/L); dissolved solids (DS, in mg/L); and electrical conductivity (EC, in mS/cm). These were measured in the field with an Icel Manaus^®^ meter (model PH–1500) with appropriate sensors. A 10 mL aliquot was collected in a new, sterile penicillin flask and used for measuring dissolved organic carbon (DOC, in mg/L) and dissolved nitrogen (N, mg/L), using a Shimadzu meter (model TOC–Vcph). Macroscopic organic debris (OD) fallen into the phytotelma was collected in a paper bag, dried for 24 hr in an oven at 80°C, and expressed in grams.

For nematode sampling, the 24 bromeliads were removed from the soil and placed in plastic bags, with care to return the water initially sucked from the phytotelma for the PCP measurements. For nematode extraction, each plant was individually defoliated and washed in tap water, collecting all the water in a 5 L bucket. The water was passed through 60 and 500 mesh sieves (250 and 25 µm opening, respectively). The resulting suspension was submitted to the method of Coolen and D’Herde (1972), with modification (no previous grinding in a blender), with centrifugation at 760.24 G for 3 min and 190.06 G for 2 min.

The 24 suspensions were reduced to a volume of 5 ml and observed entirely in Peter’s slides for nematode counting. The total abundance of nematodes and the abundance per trophic group were computed. Nematodes were assigned as plant, hyphal, or bacterial feeders, unicellular eukaryote feeders, or carnivores (Moens et al., 2006) by examination in a Nikon Eclipse^®^ microscope with Nomarski interference contrast in the 40 and 100X lenses.

### Data availability and analysis

The assays’ raw data are publicly available at https://doi.org/10.6084/m9.figshare.12925571. Unicellular eukaryote feeders were found only occasionally and plant feeders were not found. Hence, no statistics were applied to these groups. Nematode counts – total, hyphal, and bacterial feeders, and carnivores – were tested for homogeneity of variances (Cochran and Bartlett tests) and for normality of errors (Lilliefors test), at 5% probability. Since the assumptions were satisfied, nematode counts were evaluated through a two factor-ANOVA using the R language v.3.6.1 (R Core Team, 2019). The linear model accounted nematode counts as response variables, and the areas, seasons, and water PCPs as explanatory variables. The residual distribution was examined and data with residues higher than three standard deviations (|*x*| > 3 SD) were considered outliers and removed from the dataset. The pairwise comparison among least square means was done by Tukey test at 5% probability using the R language v.3.6.1. These comparisons were made by pooling data from all eight sampling dates (seasons), and by decomposing the data per season.

## Results and discussion

**Figure 2:**
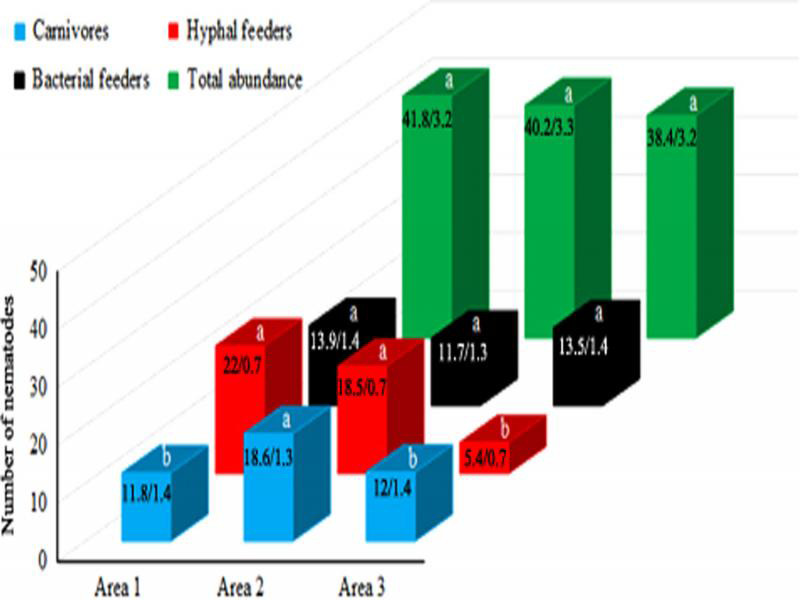
Mean number of nematodes in *Neoregelia cruenta* phytotelma in areas (1-3) with different levels of anthropic disturbance, in Restinga de Jurubatiba National Park, Brazil. For each trophic group, columns marked with the same letter are not significantly different according to the Tukey test at 5%. Numbers in the columns indicate the mean number/standard error of the mean. Values are mean of 64 sampled bromeliads.

When data from the eight sampling dates were pooled and submitted to ANOVA, there was significance for the abundance of hyphal feeders and carnivores in relation to areas (Table 2). The Tukey test indicated that the least disturbed area (3) had fewer hyphal feeders, and the area with intermediate disturbance (2) had more carnivores (Fig. 2). The three areas were statistically equivalent for total abundance and abundance of bacterial feeders.

**Table 2.** ANOVA of nematode abundance in *Neoregelia cruenta* phytotelma, in areas with different levels of anthropic disturbance, in Restinga de Jurubatiba National Park, Brazil.

Sources of variation	Statistical parameters	Area	Season	Area×season	Error	*R*^2^
Bacterial feeders	MSE^a^	123.3	363.3	393.5	94.7	0.5
	*p*-value	ns^c^	***^b^	***	–	–
Hyphal feeders	MSE	261.7	162.9	62.8	24.5	0.6
	*p*-value	***	***	**	–	–
Carnivores	MSE	771.4	570.7	469.7	96.3	0.6
	*p*-value	***	***	***	–	–
Total abundance	MSE	293.7	3877.7	1266.1	558	0.6
	*p*-value	ns	***	**	–	–

**Notes:** ^a^MSE: mean squared error; ^b^confidence levels: *5%; **1%; ***0.1%; ^c^ns: non-significant.

The greater abundance of hyphal feeder nematodes in the most disturbed areas is counterintuitive. Particularly in area 1, the bromeliads had less coverage by trees and shrubs due to wood harvesting and fragmentation of the *restinga*. We did not measure canopy coverage over the bromeliads, but we expected that less canopy coverage would lead to less impoundment of organic debris (leaves, twigs) in the phytotelma. This would negatively affect the biomass of plant litter-decomposing fungi and the abundance of hyphal feeder nematodes.

**Figure 3:**
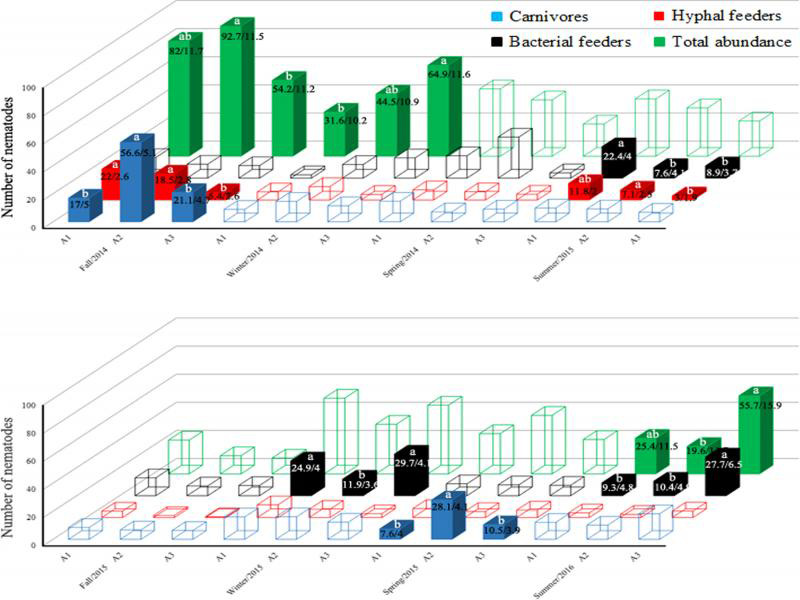
Mean number of nematodes in *Neoregelia cruenta* phytotelma in seasonal samplings in areas (A1-A3) with different levels of anthropic disturbance, in Restinga de Jurubatiba National Park, Brazil. For each sampling, empty columns indicate no significant differences among areas according to the Tukey test at 5%. Different letters indicate significant differences among areas. Numbers in the columns indicate the mean number/standard error of the mean. Values are mean of eight bromeliads sampled per season and per area.

The ANOVA of data from the eight sampling dates also revealed significance for nematode abundance (total and per trophic group) in relation to seasons, and the area x season interaction (Table 2). When we decomposed the data for the different seasons, the Tukey test indicated that areas 1, 2, and 3 were statistically equivalent for nematode abundance (total and per trophic group) in Spring 2014 and Fall 2015 (Fig. 3 and Table S1). In all other seasons, significant differences among areas were scattered among different abundance parameters. Overall, abundance patterns related to the level of AD, e.g., total nematode abundance in Winter 2014, were not confirmed in the next season or in the same season in the next year. Therefore, it seems clear that AD had no substantial effect on the nematode trophic structure in the phytotelma of *N. cruenta*.

**Table S1.** Mean number of nematodes in *Neoregelia cruenta* phytotelma in seasonal samplings in areas with different levels of anthropic disturbance, in Restinga de Jurubatiba National Park, Brazil.

Season/year	Area	BF^a^	HF	C	Total abundance
Fall/2014	1^b^	14.7a	22a	17b	82.1ab
	2	9.9a	18.5a	57a	92.7a
	3	8.9a	5.4b	21.1b	54.2b
Winter/2014	1	2.4a	5.8a	8.9a	31.6b
	2	9.7a	9.6a	14.6a	44.5ab
	3	14.4a	4a	11.5a	64.9a
Spring/2014	1	15.9a	7.2a	14.8a	48a
	2	29.3a	5.8a	7.2a	40.2a
	3	3.8a	4.2a	9.1a	22.9a
Summer/2015	1	22.4a	11.8ab	9.9a	40.9a
	2	7.6b	7.1a	9.2a	34.5a
	3	8.9b	3b	6.8a	25.1a
Fall/2015	1	12.8a	4.5a	8.4a	24a
	2	6.9a	1.5a	6.7a	12.9a
	3	7.3a	0.6a	6.3a	11.3a
Winter/2015	1	24.9a	9.1a	16a	53.9a
	2	11.9b	5.9a	16.1a	35.4a
	3	29.7a	2.7a	12.3a	48.9a
Spring/2015	1	8.5a	6.5a	7.6b	28.7a
	2	7.7a	4.3a	28.1a	41.7a
	3	7.5a	5.3a	10.5b	24.5a
Summer/2016	1	9.3b	3.3a	12.2a	25.4ab
	2	10.4b	2.3a	10.2a	19.6b
	3	27.7a	4.6a	18.2a	55.7a

**Notes:** Values are adjusted means of eight bromeliads per season and per area. For each season, values followed by the same letter in the columns do not differ by the Tukey test at 5%. ^a^BF: bacterial feeders; HF: hyphal feeders; C: carnivores; ^b^comparison of anthropic disturbance among areas: 1 > 2 > 3.

On the other hand, at times other unknown factor(s) impacted nematode abundance differently in the areas 1, 2, and 3. Major climate variables such as air temperature, rainfall, sun, and wind exposure might affect bromeliads – and their phytotelma – differently depending on the level of AD. Nonetheless, air temperature and rainfall had no impact on the nematode trophic structure in the phytotelma of *N. cruenta* (Alexandre, 2017).

Anthropic disturbance did not impact the nematode trophic structure possibly because it did not affect the phytotelma considerably. Although areas 1, 2, and 3 are distinct in their long-lasting levels of AD, the two-year monitoring of the water PCPs revealed similar values in all parameters (Table 3), with no significant difference according to the ANOVA at 5% (data not shown). This contradicts assumptions that AD reduces the impoundment of organic debris in the phytotelma, alters the energy and nutrient-cycling processes, and inevitably leads to changes in the water PCPs (Kitching, 2001; Leroy et al., 2015; Gossner et al., 2016).

**Table 3.** Descriptive statistics of physico–chemical properties of the water retained in *Neoregelia cruenta* phytotelma, in areas with different levels of anthropic disturbance, in Restinga de Jurubatiba National Park, Brazil.

	Areas					
	1^a^		2		3	
Properties	Mean (min-max)	SD	Mean (min-max)	SD	Mean (min-max)	SD
Temp (°C)^b^	21.9 (20-26)	2.1	22.1 (20-26)	2.3	21.1 (20-29)	2.6
DO_2_ (mg/L)	5.5 (2.7-8)	1.2	5.6 (3.7-7.4)	0.9	5.7 (3.2-8)	1
DS (mg/L)	64.1 (12-381)	55.1	69.8 (9-284)	54.2	80 (8-247)	56.6
EC (mS/cm)	0.09 (0.02-0.6)	0.08	0.1 (0.01-0.4)	0.08	0.1 (0.01-0.4)	0.08
pH	6.1 (4.1-8.2)	0.8	5.8 (4.5-6.8)	0.6	5.6 (3.5-8.1)	0.7
Vol (mL)	270.6 (25-845)	171.2	271.1 (20-740)	177.1	222.5 (37-910)	157.9
OD (g)	13.4 (0.3-55.8)	12.3	13.5 (1-61.4)	12.4	13.7 (1-51)	10.4
DOC (mg/L)	62.3 (13.2-210.7)	42.8	78.7 (14.9-468.3)	64.8	97.4 (15.9-574)	108.5
N (mg/L)	4.1 (0.1-17.4)	2.9	4.4 (0.1-10.8)	2.7	4.2 (0.1-12.8)	2.9

**Notes:** Values are mean of eight bromeliads sampled every season, from June 2014 through March 2016, for a total of 64 samples. ^a^Comparison of anthropic disturbance among areas: 1 > 2 > 3; ^b^Temp: temperature; DO_2_: Dissolved oxygen; DS: dissolved solids; EC: electrical conductivity; Vol: volume; OD: macroscopic organic debris; DOC: dissolved organic carbon; N: nitrogen.

Recently, Richardson et al. (2018) suggested that phytotelma fauna can be a useful bioindicator of ecosystem disturbance. This idea is supported by reports that AD impacts biomass and community structure of phytotelma invertebrates (Yanoviak et al., 2006; Khazan et al., 2015; Gossner et al., 2016). In some studies, changes in water PCPs have been linked to changes in communities of algae, archaea, bacteria, micro- and macroinvertebrates (Goffredi et al., 2011; Marino et al., 2011, 2013; Brouard et al., 2012; Carrias et al., 2012, 2014; Gossner et al., 2016; Louca et al., 2016, 2017; Kratina et al., 2017).

Our study is the first to test the hypothesis that phytotelma fauna can be a useful bioindicator of AD. The long-lasting AD affecting the *restinga* areas did not cause changes in the water PCPs. Also, AD did not affect the nematode trophic structure. Hence, it appears that the trophic structure of nematodes dwelling in bromeliad phytotelma is not a useful bioindicator of AD.

The possibility remains that AD impacts the abundance of particular nematode functional guilds, genera or species in bromeliad phytotelma. Also, higher levels of AD than those observed in areas 1 and 2 might affect the bromeliads to the extent of impacting the phytotelma nematodes. On the other hand, much needed studies on nematodes inhabiting tree and bamboo holes and pitcher plants might reveal a higher impact of AD on those nematodes, and their usefulness as bioindicators.
